# High nutrient uptake efficiency and high water use efficiency facilitate the spread of *Stellera chamaejasme* L. in degraded grasslands

**DOI:** 10.1186/s12898-019-0267-3

**Published:** 2019-12-04

**Authors:** Lizhu Guo, Jiahuan Li, Wei He, Li Liu, Ding Huang, Kun Wang

**Affiliations:** 1 Department of Grassland Science, College of Animal Science and Technology, China Agricultural University, Beijing, 100193 China; 2 GuYuan National Grassland Ecosystem Field Station, Zhangjiakou, 076550 China; 3 Department of Biology, Northwest University, Xi’an, 710069 China

**Keywords:** Ecological stoichiometry, Water use efficiency, *Stellera chamaejasme* L., Coexisting species, Degraded grassland

## Abstract

**Background:**

*Stellera chamaejasme* L. is a poisonous plant widely distributes in degraded grasslands in China. The mechanism underlying its spread remains unknown. In some degraded grasslands, *S. chamaejasme* has gradually replaced previous dominant species, such as *Leymus chinensis*, *Stipa krylovii*, *Artemisia eriopoda* on typical steppes. Apart from its unpalatability by livestock, we hypothesized that the survival strategy (nutrient uptake and water use efficiency) of *S. chamaejasme* in degraded grasslands could be distinct from other coexisting species in the community. Recently, ecological stoichiometry has been suggested as a new approach for studying the demand for natural resources of plants in a changing world, and the leaf carbon isotopic composition (δ^13^C leaf) as a rapid and effective high throughput phenotyping method for water use efficiency (WUE), both of which can reveal the survival and adaptive strategies of plants. Therefore, in this study we aimed to fill the knowledge gap concerning ecological stoichiometry in the leaf, stem, and root of *S. chamaejasme* and its surrounding soil on grasslands with different degrees of degradation, and comparing the leaf nutrient content and δ^13^C of *S. chamaejasme* with the coexisting species (*L. chinensis*, *S. krylovii*, *A. eriopoda*) in the communities. Toward this goal, we conducted a field survey in which plants and soils were sampled from four different degraded grasslands on typical steppes in China.

**Results:**

Our results showed that there is no significant difference of carbon content (C%) and nitrogen content (N%) in leaves of *S. chamaejasme* in different degraded grasslands, and all element contents and element ratios in stems did not differ significantly. Meanwhile, ecological stoichiometry of *S. chamaejasme* is distinct from the coexisting species, with low C%, high N% and phosphorus content (P%) in the leaf, indicating high nutrient uptake efficiency of *S. chamaejasme* in nutrient-poor environments like degraded grasslands. Additionally, *S. chamaejasme* showed significant higher WUE than other species.

**Conclusions:**

Our results indicated that high nutrient uptake efficiency and high WUE of *S. chamaejasme* might together contribute to the spread of *S. chamaejasme* in degraded grasslands.

## Background

Covering 30–40% of the earth’s terrestrial surface, grasslands are one of the most widespread vegetation types on the earth. Of these, 8.9 million km^2^ are situated in Asia, with nearly 4 million km^2^ occurring in China [1]. *Leymus chinensis* steppe is widely distributed in the Eurasia steppe zone and is one of the dominant steppe types in the Inner Mongolian Plateau [2]. Grasslands play an important role in livestock farming and environmental conservation. However, data show that 90% of all natural grasslands in China have degraded to different extents, and the degradation is becoming wider through overgrazing, cropland misuse and unregulated collection of fuel and medicinal plants [3–5]. Grassland degradation is often accompanied by the increase in the area of poisonous plants (while herbage mass loss, especially *L. chinensis*). Currently, the increasing attention given to poisonous plants on degraded grassland has been matched by a growing body of literature [6–9] on this subject, which tends to emphasize another dimensions of the grassland degeneration problem. *Stellera chamaejasme* L., one of the most popular poisonous plants, is widely distributed throughout the degraded grasslands in China [10]. The whole plant of *S. chamaejasme* is poisonous and its roots and pollens are most toxic, therefore livestock may be poisoned simply by inadvertently inhaling the pollen while grazing [8]. *S. chamaejasme* is also a companion species in normal grassland but the communities dominated by *S. chamaejasme* often result in severely degraded grassland. In our research area, we found some dominant palatable forages (*L. chinensis*, *Stipa krylovii*, *Artemisia eriopoda*) have become fewer in communities, while *S. chamaejasme* has been increasing over time.

Grassland degradation caused by overgrazing is often accompanied by changes in vegetation and soil [5]. The change of vegetation, especially the change of species in community [11, 12], is not only due to the selective consumption by herbivores [13, 14], but also related to the different responses of different species to environmental fluctuations [15–19]. Meanwhile, soil nutrients variation, one of most useful soil response indicators, will affect the nutrient uptake of plant species, thus further affecting their survival and growth. Although the increase of *S. chamaejasme* in degraded grassland is mostly related to its unpalatability by livestock, it may also be associated with its distinct response to soil nutrients variation after grassland degradation. Ecological stoichiometry represents an organism’s demand for natural resources and connects different levels of biogeochemical cycling [20, 21], mainly by scaling up carbon (C), nitrogen (N) and phosphorus (P) [22, 23]. It provides a powerful tool for ecologists to study nutrient cycling [22, 23] and understand the demand for natural resources of different species in communities, and their responses to grassland degeneration.

On the other hand, water is a common limiting factor for plant growth in the vast grassland in the arid and semi-arid northern China, and soil water content has been decreasing due to less vegetation cover on soil surface in degraded grasslands [5, 24]. Fischer initially proposed the use of dry matter produced by evaporating unit water as a term for transpiration efficiency [25]. With the development of technology, leaf carbon isotopic composition (δ^13^C leaf) has been suggested to serve as a rapid and effective high throughput phenotyping method for water use efficiency (WUE) in both C3 and C4 species [26]. This is because WUE and δ^13^C leaf are correlated through their relationships with intercellular to ambient CO_2_ partial pressures (Ci/Ca) [27, 28]. Therefore, high WUE is considered to be a contributing feature of plant growth and production in arid and semi-arid environments, especially facing water shortage in degraded grasslands.

In recent years, a wide range of studies [29, 30] have focused on typical dominant plants in natural grasslands. However, there has been little attention on the ecological stoichiometry of poisonous plants. *S. chamaejasme* has been recently of increasing interest to grassland researchers [31–34], but there is little knowledge about its ecological stoichiometry. Although there is hardly any disturbance from herbivores to *S. chamaejasme*, we suppose that the distinct response of *S. chamaejasme* to grassland degeneration may help it outcompete other coexisting species. Species’ variation in stoichiometry characteristics and WUE allowed us to shed light on why *S. chamaejasme* could spread widely in degraded grasslands in China. Therefore, we mainly focus on two questions in our study: first, how does the stoichiometric characteristics of different organs of *S. chamaejasme* change with grassland degradation; second, does *S. chamaejasme* perform distinctly from other co-existing species in degraded grasslands?

## Results

### C, N and P contents and C:N:P characteristics of soil and *S. chamaejasme* in degraded grassland

The average values of topsoil (0–10 cm) C, N, P in degraded grassland are 3.60%, 0.39%, and 0.07%, respectively (Table 1). There was a clear trend of reduced vertical distribution of the three nutrient concentrations in the soil, which decreased with the deeper soil layers. The C/N ratio increased gradually with an increase in soil depth, whereas the C/P and N/P ratio decreased stepwise. In *S. chamaejasme*, the leaf exhibits the highest C content (38.64%), N content (4.51%) and P content (0.24%), followed by the stem (37.33%, 2.37% and 0.09%, respectively) and root (36.60%, 2.19% and 0.07%, respectively). Moreover, the leaf has the lowest C/N, C/P and N/P ratios, but the root has the highest values in all three.

**Table 1:** Statistics of C, N and P contents and C:N:P characteristics (Mean ± SE) of all soil and *S. chamaejasme* samples

	C%	N%	P%	C/N	C/P	N/P
Leaf	38.64 ± 2.39	4.51 ± 0.31	0.25 ± 0.05	8.6 ± 0.4	161.6 ± 35.1	18.8 ± 3.5
Stem	37.33 ± 2.32	2.37 ± 0.14	0.09 ± 0.02	15.8 ± 0.8	427.1 ± 119.8	27.0 ± 7.0
Root	36.60 ± 3.06	2.19 ± 0.25	0.07 ± 0.03	16.8 ± 1.5	606.9 ± 245.3	35.7 ± 12.9
Soil (0–10 cm)	3.60 ± 1.55	0.37 ± 0.10	0.07 ± 0.01	9.3 ± 1.7	53.3 ± 20.1	5.6 ± 1.2
Soil (10–20 cm)	2.87 ± 1.21	0.28 ± 0.11	0.06 ± 0.01	10.3 ± 2.5	50.2 ± 15.9	5.0 ± 1.3
Soil (20–30 cm)	2.08 ± 1.14	0.20 ± 0.10	0.05 ± 0.01	10.7 ± 4.2	40.3 ± 16.0	4.0 ± 1.5
Soil (30–40 cm)	1.74 ± 1.02	0.16 ± 0.09	0.04 ± 0.01	11.3 ± 5.2	39.2 ± 21.0	3.5 ± 1.5

### C, N and P contents and C:N:P characteristics of *S. chamaejasme* under different degraded degree of grassland

**Fig. 1:**
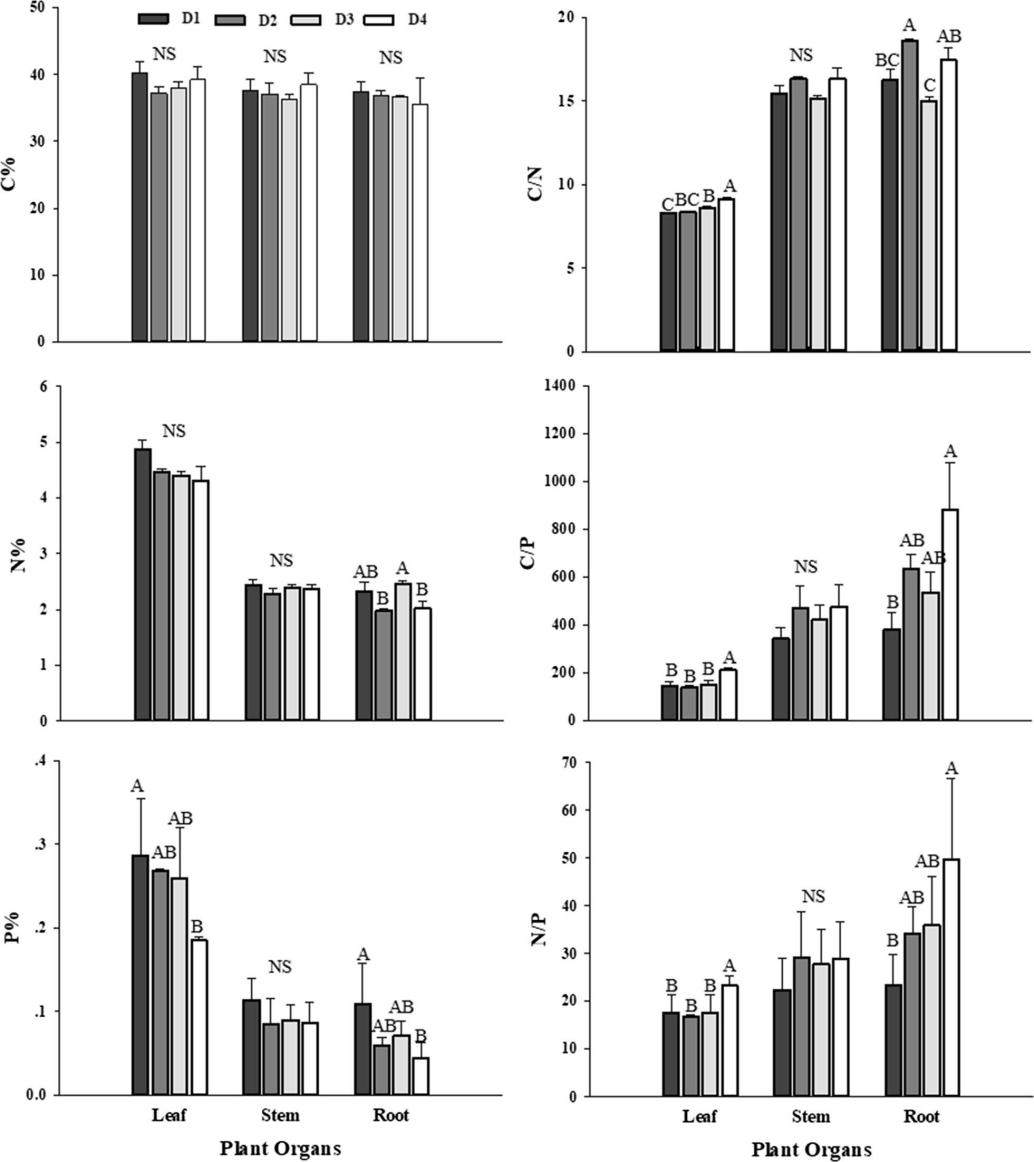
Stoichiometric characteristics (Mean ± SE) of C, N, P, C/N, C/P and N/P ratios in *S. chamaejasme* as affected by degradation degree. Values designated by different capital letters were significantly different among the four degraded grasslands levels (P < 0.05). The same applies below

There was no significant difference of *S. chamaejasme* leaf C% and N% in the four sample plots, and lowest leaf P% in D4 plot made its element ratios (C/P and N/P) highest among all plots (Fig. 1). For stems, all element contents and element ratios did not differ significantly. However, for roots, only C content did not change significantly. Moreover, the C% content in three organs of *S. chamaejasme* (leaf, stem and root) were no difference (P > 0.1) in all sample plots, but the concentrations of N and P were higher in leaves than in stems and roots, and therefore three element ratios of leaves were lower than those of other two organs.

### Soil nutrients and C:N:P characteristics under different degrees of degraded grassland

**Fig. 2:**
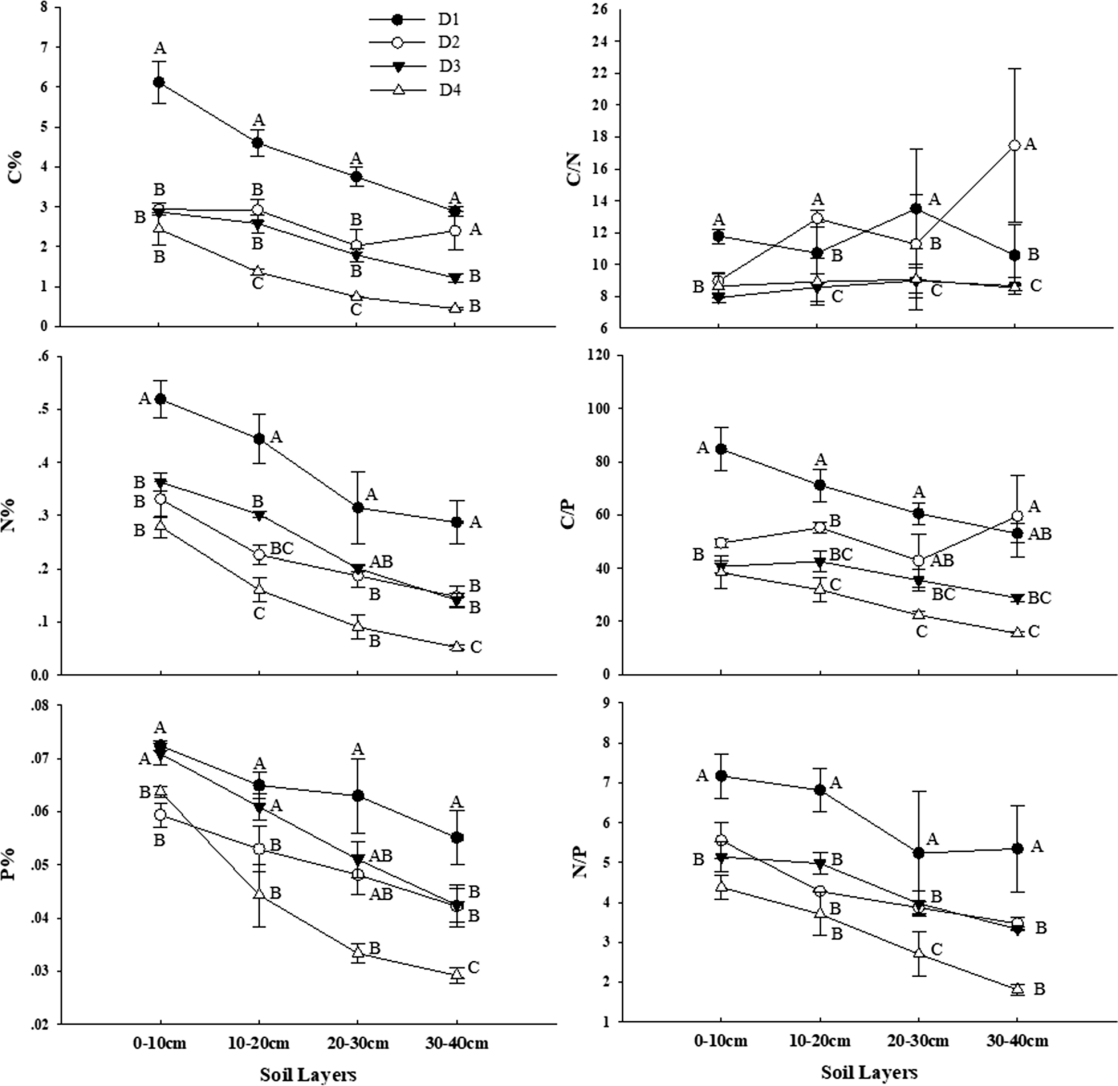
Stoichiometric characteristics (Mean ± SE) of C, N, P, C/N, C/P and N/P ratios in soil as affected by degradation degree

Figure 2 showed that C%, N%, and P% in soil decreased continuously with grassland degradation, however, there was little difference in nutrient contents between D2 and D3. Similarly, three elemental ratios had a general downward trend, with one exception, the C/N ratio of D2 in 10–20 cm and 30–40 cm soil layers were highest. What is more, topsoil C content (0–10 cm) in D1 grassland was highest among four sample plots, and the rest of the plots showed little difference. Soil C content in rest soil layers decreased significantly as soil depth deeper. Except that the C/P ratio of D2 changed irregularly, soil N%, P% C/P ratio, as well as N/P ratio of all different degraded grasslands became lower with soil depth increase. Soil C/N ratio of D1 and D2 did not show a clear trend, but D3 and D4 kept relatively stable with the increasing of depth of soil.

### Comparisons of stoichiometric characteristics and carbon isotope composition (δ^13^C) of leaf between *S. chamaejasme* and other species

**Fig. 3:**
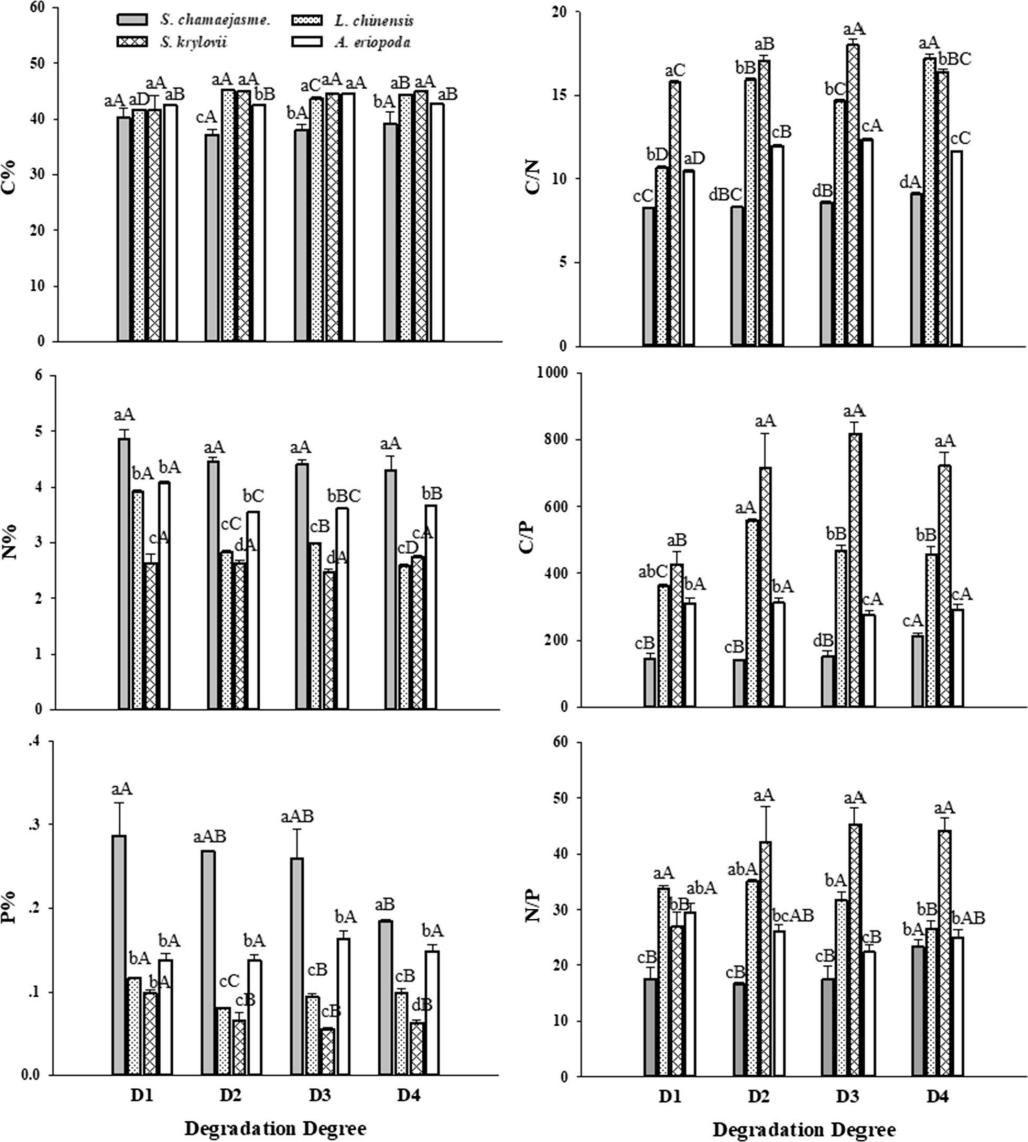
Comparison of stoichiometric characteristics of leaves (Mean ± SE) between *S. chamaejasme* and three coexisting species (*L. chinensis*, *S. krylovii* and *A. eriopoda*). Values designated by different lowercase letters were significantly different among four species. Different capital letters indicate a significant difference among the four degrees of degradation, respectively (P < 0.05)

When compared with the other three coexisting species (*L. chinensis*, *S. krylovii* and *A. eriopoda*), *S. chamaejasme* showed some unique stoichiometric characteristics (Fig. 3). *S. chamaejasme* showed lower C concentration than other three species, although there was no significant difference of C% among the four species in D1 plot. However, N concentrations of *S. chamaejasme* (> 4%) was larger than that of others (< 4%), and P concentrations was more than 1.2 times of that of others. N% and P% of *S. krylovii* were lowest, making its C/N, C/P and N/P values highest among nearly all plots. Moreover, the C% and N%, of *S. chamaejasme* were no significant difference among four degraded grasslands, but P% decreased significantly in D4 plot, and therefore values of C/P and N/P of D4 were the highest. Interestingly, all indexes of *L. chinensis* changed significantly, while other species have some indexes that different insignificantly, like C% and N% for *S. chamaejasme* and *S. krylovii*, P% for *A. eriopoda*. In addition, carbon isotope composition (δ^13^C) of *S. chamaejasme* was the highest among four species in all plots (Table 2). The result also showed that δ^13^C of four species change significantly with grassland degradation, but not in a similar trend.

**Table 2:** Comparison of leaf carbon isotope composition (δ^13^C) (Mean ± SE) between *S. chamaejasme* and the three coexisting species (*L. chinensis, S. krylovii and A. eriopoda*) (‰)

	*S. chamaejasme*	*L. chinensis*	*S. krylovii*	*A. eriopoda*
D1	− (23.22 ± 0.06)aD	− (27.96 ± 0.02)bD	− (27.99 ± 0.03)bC	− (28.20 ± 0.06)cC
D2	− (22.99 ± 0.04)aB	− (26.29 ± 0.09)bC	− (27.42 ± 0.04)cB	− (28.38 ± 0.05)dD
D3	− (22.70 ± 0.02)aA	− (26.08 ± 0.04)bA	− (26.83 ± 0.01)cA	− (26.02 ± 0.03)bA
D4	− (23.10 ± 0.05)aC	− (26.20 ± 0.04)bB	− (26.89 ± 0.04)cA	− (27.76 ± 0.02)dB

Values designated by different lowercase letters were significantly different among four species. Different capital letters indicate significant difference among the four degrees of degradation, respectively (P < 0.05)

## Discussion

### C, N and P contents and C:N:P characteristics of *S. chamaejasme* under different degree of grassland degradation

No significant differences were found in concentration of two elements (C% and N%) in *S. chamaejasme* leaf among the four sampling plots, and a similar trend existed in organ-specific C. As a typical perennial plant, *S. chamaejasme* allocates more biomass to root, as the root plays important roles not only in accumulation of reserves for regeneration, but also in defensive compounds of roots to avoid being eaten by livestock [35–38]. In contrast to C, plant N and P concentrations vary significantly among different organ types, which can be ascribed to the differences in the structure and physiology of organs [39]. Generally, leaves perform a number of physiological functions (such as photosynthesis, respiration, and water utilization) that are vital to plant survival and productivity in particular under extreme conditions such as degraded environments, and as such they require higher levels of nutrients to ensure normal operation of these functions. Meanwhile, it is well known that the main function of the stem is transporting nutrients between leaf and root, whereas leaves and roots are the organs responsible for resource uptake by plants [40]. Therefore, it is not surprising that no significant difference in elements and ratio of elements were found in stems. Contrary to elemental trends, elemental ratios of the root always showed the highest values, mainly because of the low level of N% and P% in the root, but equal C content to the leaf and the stem. C/P ratio and N/P ratio of *S. chamaejasme* leaf remained relatively stable except for D4, totally because the lowest P% of D4. Besides, there was a significant increase of C/N ratio in the leaf and C/P and N/P ratios in the root with degraded sampling places. The reason why C/N and C/P increased was that C% was relatively stable, while N% and P% decreased to a certain extent during grassland degradation.

### Soil nutrients and soil C:N:P characteristics under different degrees of grassland degradation

Soil elements respond more slowly than vegetation aboveground biomass and cover to grazing, and are thus often seen as reliable indicators of grassland degradation [5]. Similar to previous studies, soil nutrient content decreased with the increase of soil depth, and also decreased with the aggravation of grassland degradation at the same soil depth [41–43]. With the highest and lowest values occurring in the D1 and D4 sites respectively, no great difference was found between D2 and D3 in our research areas. This could be related to complex responses of soil properties to livestock grazing in different degraded grasslands. Consumption of aboveground biomass by livestock and reduction of the carbon pool in aboveground biomass and litter could lead to direct carbon loss in soil [44]. Meanwhile, livestock take up nutrients across grasslands, but release them in the form of dung and urine, and local residents collect dung as fuel for heating or as organic fertilizers for planting, which leads to further nutrient losses in degraded grasslands [5, 45]. Our findings demonstrate that compared with soil N declines significantly (1.82 times between highest and lowest value in 0–10 cm soil layer and 5.16 times in 30–40 cm soil layer), P content did not change much (around 1.5 times among all soil layers). The result was consistent with the findings in Qinghai–Tibet Plateau grassland, which found that the decrease of total N% in soil invaded by *S. chamaejasme* was higher than that of total P% [46]. Therefore, N might be the more limiting nutrient element in our research area, which is similar as reported in other arid and semi-arid regions [47].

The ration of C/N in soil was recognized as the essential factor that influences the equilibrium of C and N cycling, which is determined by activity of microorganisms and by plant absorption [48, 49]. Both mineralization and nitrification rates increase as the C/N ratio of soil organic matter is reduced. The soil C/N ratio in our study area showed a downward trend in the same soil layer as degradation intensified, meaning that grazing-induced grassland degradation reduces the soil C/N ratio and stimulates soil microbial activity [50, 51]. There is one exception, D2 in 10–20 cm and 30–40 cm soil layers has a higher C/N ratio than that of D1, which come from different reason, for D2 in 10–20 cm soil depth contain less nitrogen, while the carbon of 30–40 cm of D2 was more than that of D1. Likewise, C/P and N/P ratios decreased dramatically with the soil depth and degradation degree. Through analyses of C/P and N/P ratios, we found that C and N content varied a great deal, but P content kept relatively stable in soil. That is because unlike the soil C and N, the weathering of the parent material, which is located at the bottom of the soil profile, provides the major source of available soil P [52].

### Comparisons of stoichiometric characteristics and carbon isotope composition (δ^13^C) of leaf between *S. chamaejasme* and other species

Due to the comprehensive influence of different factors, the stoichiometric changes of N and P in some areas do not conform to the rules drawn from the national large-scale range. Comparing the value of the leaf with published data [53–55], it can be seen that four species in our research differed greatly from published result. High N and low P in three species (*L. chinensis*, *S. krylovii* and, *A. eriopoda*), resulted in low C/N ratio, high C/P ratio and N/P ratio. The reason may be that the content and ratio of nutrients in plants can be constrained by nutrient supply in soil, and the content of soil N is high and soil P is low in our study area, therefore generating this difference.

Plant nutrient concentrations differ widely among species [56]. The fact that N concentration and P concentration among the four species in four sample sites differed significantly supported this assertion. When compared with coexisting species, *S. chamaejasme* leaf has lower carbon content in 3 of 4 plots. The low leaf C levels may be attributable to the plants need to consume additional energy to form poisonous secondary metabolites in order to avoid being eaten by livestock, which leads to increased metabolic costs [57]. It is worth noting that N concentrations of *S. chamaejasme* kept high value (> 4%), and was greater than that of others (< 4%); besides, P concentrations was higher either. Both mean that high nutrient uptake efficiency of *S. chamaejasme* in nutrient-poor environments like degraded grasslands. As described in previous experiments on *S. chamaejasme*, although *S. chamaejasme* did not absorb more N than other species, nearly 80% of nitrogen uptake was distributed to aerial parts [58], which may be the reason why *S. chamaejasme* leaf contains high N. High P in *S. chamaejasme* may be correlated positively with its short growth cycle in the field (from 20th May to 10th July), because faster growing tissues need relatively more P-rich RNA to support rapid protein synthesis [59]. Alternatively, these species-specific responses may stem from different performance when facing livestock in the degraded grassland [60, 61]. The lower values in leaf N and P concentrations of three co-existing species might therefore stem from its decreasing biomass to compete with *S. chamaejasme* for soil resources as the ecosystem becomes more infertile. The three element ratios (C/N, C/P and N/P) in *S. krylovii* were highest among four species, and it is mainly because N and P concentrations in *S. krylovii* were the lowest. It is known that when plant C/N ratio becomes small, the decomposition rate of the residue becomes faster [62]. Due to the input of large amounts of high quality aboveground litter (high N%) without livestock disturbance, low C/N ratio may help *S. chamaejasme* to create islands of fertility. Previous research found that *S. chamaejasme* significantly increased surface soil (0–15 cm) organic matter [63], which is similar with our results. Generally, it is not uncommon that using N/P ratios of plant biomass as indicators of N or P limitation in various studies [64–66]. A threshold of N/P ratio for N and P limitation used in our study comes from an experimental result of China typical steppe [2], which indicates the species is N limited if N/P is < 21, while P limited if N/P > 23. The low N/P ratio in *S. chamaejasme* might imply that its growth is relatively restricted by N (mean N/P ratio = 18.8), however, the rest of the species (*L. chinensis* 31.8, *S. krylovii* 39.6, and *A. eriopoda* 25.7) could be P limitation. Understandably, the demand for high N content in leaves of *S. chamaejasme* causes its growth to be limited by N. However, it is interesting that in our study whether the low N content in soil or the N-limited growth of *S. chamaejasme*, high leaf N level was detected in *S. chamaejasme*. It is not consistent with high soil N%, and high leaf N% in other studies [67]. Our findings may be explained partly by the Stability of Limiting Elements Hypothesis, which suggests that nutrients required at a high concentration by plants, and which are most frequently considered to be limited in environments, should be less sensitive to environmental factors [23]. Therefore, high leaf N content makes *S. chamaejasme* less sensitive to N deficiency in degraded grasslands.

Average leaf δ^13^C of *S. chamaejasme* was − (23.01 ± 0.20) ‰, and *L. chinensis* − (26.63 ± 0.81) ‰, *S. krylovii* − (27.28 ± 0.49) ‰, *A. eriopoda* − (27.59 ± 0.98) ‰, which were in the range of δ^13^C of Chinese plants (− 33.50‰ to − 22.00‰) [68]. Leaf δ^13^C of four species had irregular variations with increasing degradation, however, *S. chamaejasme* always showed the highest value than other species in all plots. Generally, due to its positive relationship with water use efficiency (WUE), high δ^13^C is considered as a trait contributing to the successful growth and production of species in arid and semiarid environments [27, 28]. Our result indicated that the WUE of *S. chamaejasme* was significantly higher than other three co-existing species, and the deep root system of *S. chamaejasme* can be used to explain its efficient WUE, thus ensuring its competitive advantage in degraded grasslands.

## Conclusions

In our study, we found that there is no significant difference of C% and N% in *S. chamaejasme* leaf in different degraded grasslands, and all element contents and element ratios in stems did not differ significantly. Compared with other species, *S. chamaejasme* leaf was characterized by low C% and high N%, P%, and high N%, P% means its high nutrient uptake efficiency in degraded grasslands when soil nutrients become less. Finally, *S. chamaejasme* always showed the highest value of δ^13^C than other species in all plots, indicating that the WUE of *S. chamaejasme* was significantly higher than others in degraded grasslands. In total, high nutrient uptake efficiency and high WUE could enable *S. chamaejasme* to survive and grow better than the other species, leading to its spread in degraded grasslands.

## Methods

### Study area

**Fig. 4:**
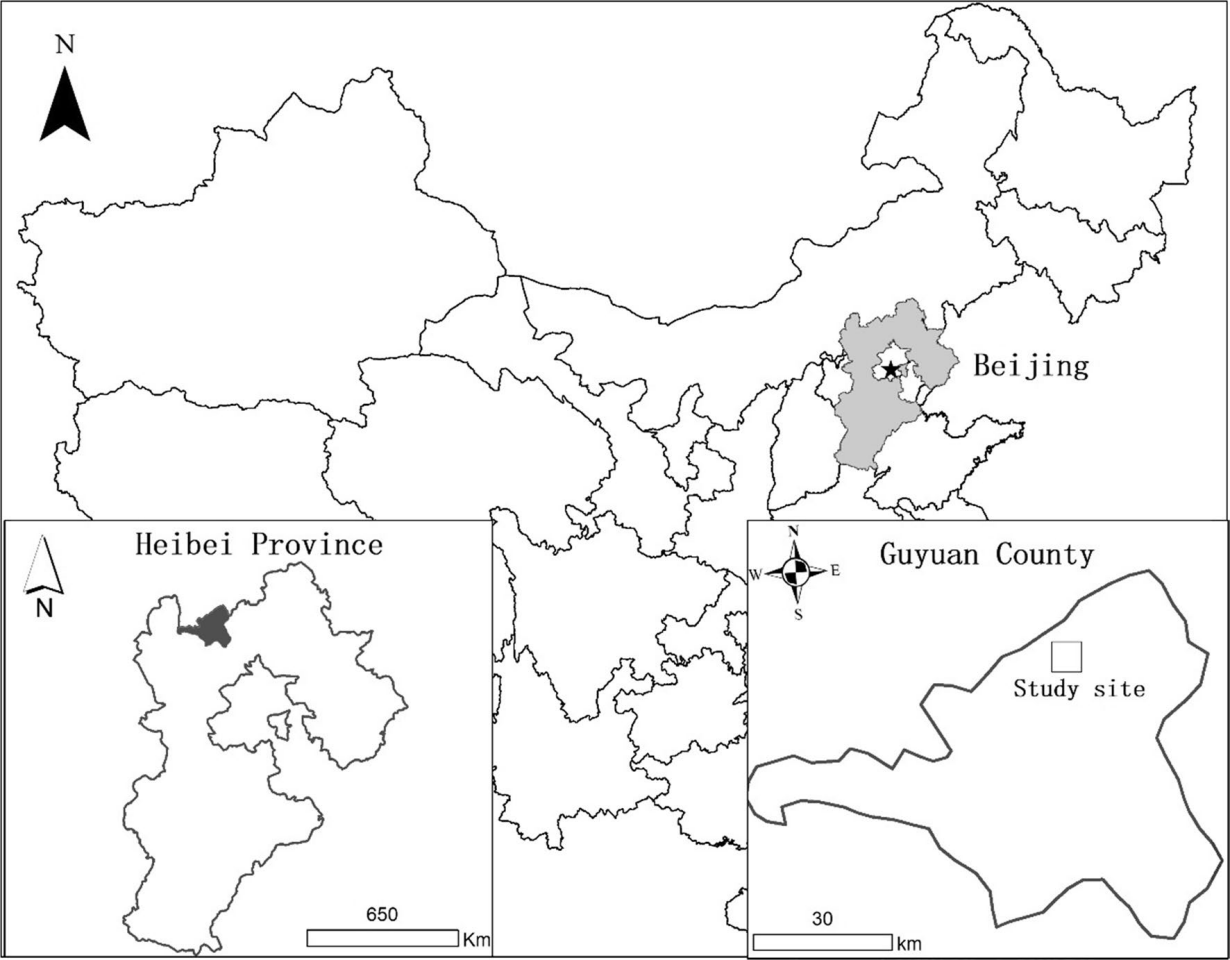
Map of the study site. Map created with free version of carto (https://carto.com/) by using 1:400 million dataset in China national fundamental geographic information (http://www.data.ac.cn/)

The research was conducted at the National Field Station for Grassland Ecosystems in Guyuan County (latitude 41° 46′ N, longitude 115° 40′ E, elevation 1430 m), Hebei Province, China (Fig. 4). The area has a semi-arid continental monsoon climate with a frost-free period of 80–100 days. The annual mean precipitation is approximately 430 mm (ranging from 350 to 450 mm), and approximately 80% of the precipitation is concentrated in the growing season between June and September. The annual mean air temperature is 1.4 °C. The minimum/lowest monthly mean air temperature is − 18.6 °C in January, and the maximum is 21.2 °C in July. *L. chinensis* is the dominant species of typical steppe, which is the main type of grassland in the local area, and the soil is Calcic-orthis Aridisol [69].

### Selection of sampling plots

Four sampling plots (at least 10 ha each plot) with different degrees of degradation (D1–D4) were selected according to their community characteristics and soil physical and chemical properties. Meanwhile, four targeted species (*S. chamaejasme*, *L. chinensis*, *S. krylovii* and *A. eriopoda*) all appeared in four selected plots. With grassland degradation, *L. chinensis* in grassland decreases dramatically. *S. krylovii* and *A. eriopoda* show less decrease than *L. chinensis*, however, *S. chamaejasme* tends to flourish. To be specific (Table 3), plot D1 site had best performance among four main indexes of community feature; while D2 had significantly less biomass and species number and coverage than D1, but its proportion of grasses decreased slightly; D3 was similar to D2, but the greatest difference was that its proportion of grasses was less than D2; D4 showed higher number of species, coverage than D2 and D3 unexpectedly, mainly due to the larger proportion of annual or poisonous weeds in the community. Moreover, surface soil features of different degraded grassland was shown here (Table 4), and it displayed clearly that physical and chemical properties of soil decreased with the degradation of grassland.

**Table 3:** Plant community features in different degraded grassland (Mean ± SE)

	Number of species	Total biomass (g/m^2^)	Ratio of grasses biomass (%)	Coverage (%)
D1	37	174.74 ± 44.83	88.17 ± 18.04	61.87 ± 17.32
D2	23	133.85 ± 27.23	84.18 ± 11.34	31.63 ± 5.28
D3	23	126.14 ± 9.17	69.79 ± 2.61	36.55 ± 7.69
D4	46	144.96 ± 39.04	48.05 ± 12.26	50.54 ± 10.46

**Table 4:** Surface soil features in different degraded grassland (Mean ± SE)

	Water content (%)	pH	Electrical conductivity (μS/cm)	Total carbon (mg/g)	Total nitrogen (mg/g)	Hydrolytic nitrogen (mg/kg)	Total phosphorus (mg/g)	Available phosphorus (mg/kg)
D1	17.95 ± 3.26	8.30 ± 0.10	273.93 ± 24.57	96.68 ± 16.88	5.62 ± 0.80	367.62 ± 66.08	1.74 ± 0.31	1.42 ± 0.37
D2	11.48 ± 1.19	8.65 ± 0.11	213.57 ± 12.42	50.53 ± 6.37	2.96 ± 0.56	178.18 ± 32.32	0.89 ± 0.31	0.88 ± 0.16
D3	14.39 ± 5.90	8.24 ± 0.03	246.07 ± 7.92	50.45 ± 5.81	3.78 ± 0.10	229.25 ± 14.86	1.93 ± 0.29	1.34 ± 0.39
D4	7.76 ± 1.23	9.39 ± 0.83	672.00 ± 55.50	25.54 ± 2.39	1.53 ± 0.96	116.67 ± 66.83	0.41 ± 0.20	1.03 ± 0.31

### Collection of samples

Field measurements were conducted in June 2017, which was the vigorous growth stage for *S. chamaejasme*. We randomly took 20 individual *S. chamaejasme* samples in each sampling plot, then the leaf, stem and root samples from the whole plant were carefully separated and cleaned, and oven-dried at 65 °C to a constant mass. Meanwhile, 20 plants of *L. chinensis*, *S. krylovii* and *A. eriopoda* with good growth features were selected too, and the leaves were mixed into a composite sample. Afterwards, they were ground into fine powder for testing the content of elements (C%, N% P%) and carbon isotope composition (δ^13^C) of each plant. In addition, five soil samples (0–40 cm in depth) were collected from each site, and each sample was thoroughly mixed with four subsamples and air-dried. Roots in the soil were removed by hand and sieved through a 100-mesh sieve. Then, the soil was divided into three subsamples for analyzing soil organic carbon, total nitrogen and total phosphorous.

### Measurement and analyses

Total carbon and nitrogen concentration of plant and soil samples were determined sequentially by a FLASH 2000 elemental analyzer (Thermo Fisher Scientific, MA, USA). Total phosphorus of plant and soil samples, and soil available phosphorus were used in molybdenum–antimony anti-spectrophotometric method. Soil available nitrogen was tested by Conway Method. Soil moisture measurement was in drying method, and soil pH and electrical conductivity were measured by PC2700 Desktop pH/Conductivity Measuring Instrument (Thermo Fisher Scientific, MA, USA). Leaf δ^13^C content was analyzed by Isoprime 100 (Elementar Analysensysteme, Germany). All analysis of samples was conducted in the laboratory of the China Agricultural University, Beijing.

One-way analysis of variance (ANOVA) was used to test significant differences in C%, N%, P%, C/N ratio, C/P ratio, N/P ratio and δ^13^C between degradation degrees or between plant species, respectively, and then followed by multiple comparison by Duncan’s post hoc test or the Games–Howell test for heterogeneous variances.

## Data Availability

All the data were summarized in the manuscript itself. Please contact the corresponding author regarding any additional queries related to the dataset generated and analyzed during the current study. The datasets in this study are available from the corresponding author on reasonable request.

## Abbreviations

C: carbon
N: nitrogen
P: phosphorus
WUE: water use efficiency
δ^13^C: carbon isotopic composition
CO_2_: carbon dioxide
Ci: intercellular carbon dioxide
Ca: ambient carbon dioxide
